# Risks related to the lack of lubrication on surface integrity in drilling

**DOI:** 10.1016/j.heliyon.2019.e01138

**Published:** 2019-01-17

**Authors:** Mathieu Girinon, Habib Karaouni, Ugo Masciantonio, Fabien Lefebvre, Erwan Jourden, Frédéric Valiorgue, Joël Rech, Eric Feulvarch

**Affiliations:** a SAFRAN Tech, rue des Jeunes Bois, 78772, Magny-les-Hameaux, France; b Univ. de Lyon, ENISE, LTDS, UMR5513 CNRS, 58 rue Jean Parot, 42023, Saint-Etienne, France; c CETIM, 52 avenue Félix Louat, 60300, Senlis, France; d AREVA NP, 10 rue Juliette Récamier, 69006, Lyon, France

**Keywords:** Mechanical engineering

## Abstract

Drilling processes can significantly affect the surface integrity of metallic components. Thus, the control of the drilling process is of great interest for industry to ensure a satisfactory surface behavior. The understanding of such a process is complex because the hole is confined. Therefore, the physical phenomena are not easily observable during a drilling operation. The aim of this article is to present an experimental investigation on the effect of the drilling conditions for the machining of an austenitic stainless steel 316L. Three cases of lubrication are studied: internal coolant, external coolant and dry. The objective is to identify the mechanical and thermal contributions on surface integrity. In this study, the influence of the lubrication conditions is first characterized during the drilling operation (temperatures around the drilled surface, forces…). Then, the effect of lubrication on the surface integrity after drilling is clearly highlighted by analyzing the hole diameters, the drill deflections, the roughness, the residual stresses, the hardness and the microstructure.

## 1 Introduction

In industry, the lifetime of critical mechanical components must be controlled. It is directly linked to the surface integrity that directly depends on the thermo-mechanical history during the manufacturing processes. In most machining processes, the cutting zone can be experimentally observed. In drilling, the area of material removal is confined in the hole and is not easily accessible for experimental analysis. Because of this difficulty, the drilling process is not studied as completely as turning processes even if it is widely used for the manufacturing of critical components (aeronautic, nuclear, medical fields).

During a machining operation, the lubrication conditions have an important effect [1]. Several studies present the lubrication influence in machining. Sankar and Choudhury [2] performed a study about machining with dry compressed air, flood and minimum quantity cutting fluid cooling techniques. Minimum quantity cutting fluid is the best condition to reduce wear and surface roughness. Abdel-Aal *et* al [3] studied the thermal influence on tool wear during machining. These different studies highlight the need for a good understanding of the lubrication effects on the machined part before reducing it.

Several works study the influence of lubrication during drilling [4, 5]. Bhowmick and Alpas [6] focus on the contact between the drill tool and the removed material during the drilling of aluminum alloys. Kuram et al [7] investigate the influence of oil types (mineral or vegetable, raw and refined) on the thrust force and surface roughness for the drilling operation of a 304L steel. Zeilmann et al [8] analyze external cooling during a drilling operation and characterize the drill wear, the roughness and the affected layer on the final workpiece under three conditions (emulsion, MQL with pecking cycle and dry with pecking cycle). The average plastic deformation is highest in dry conditions whereas the MQL technique is just below emulsion. Zeilmann and Weingaertner [9] performed a drilling test in Ti6Al4V with thermocouples located 0.2 mm from the final hole surface. They showed that internal MQL significantly reduces the temperature in the machined component compared to dry drilling whereas external MQL does not lead to satisfactory results. As a conclusion, lubrication is of great importance in drilling but no study observed its influence at the same time during the hole generation process and after drilling.

In addition to the thermal effects, the preparation of cutting edges can play an important role on surface integrity as studied by Bordin et al. [10] in dry drilling configuration. A three-edge preparation is studied (sharpened, polished with abrasive brushes, drag finished). In the case of the polished cutting edge, a lower temperature (thermocouples located at 0.1 mm depth from the hole surface) leads to a lower affected layer. In another study [11], the thermocouple is embedded in the drill point near the cutting edge. A predictive model allows determining the temperature in the workpiece, the diameter deviation and the contribution of the drill expansion and workpiece constriction. These studies do not focus on the lubrication conditions but deal with the link between the temperatures in drilling and the quality of the hole.

Several studies highlight the influence of the lubrication conditions during drilling on the roughness or affected layer from the microstructure point of view. Unfortunately, no study analyzes what happens when going from the cutting indicators to the impact on the surface integrity for different lubrication conditions.

In this article, the evolution of temperatures in several points, drilling forces, chip, contact surface, deflection and radial force are investigated for each lubrication condition studied. Thus, the thermal and mechanical phenomena that occur in drilling can be identified. The evolution of the drilling conditions according to depth is also studied. Moreover, the quality of the hole for each lubrication condition is analyzed (hole diameter, microstructure of the drilled surface, microhardness and residual stresses). The link between the importance of mechanical or thermal phenomena and the type of residual stresses is established. The evolution of the quality of the hole is also studied and limits in dry conditions are presented.

The aim of this article is to study the surface integrity and thus the residual stresses induced by drilling. To avoid a dependency on the previous state of the material and to identify only the phenomena induced by the drilling operation, an annealing treatment is performed on the sample before drilling to remove bulk residual stresses due to different previous processes (forging and machining).

## 2 Experimental

The material studied is a 316L austenitic stainless steel. The drilled sample has a 60 mm diameter and a 36 mm thickness corresponding to the studied through-hole. The drill bit of 12 mm diameter is made of carbide and is coated in TiAlN-TiN.

Three lubrication cases are studied:

• Internal coolant, emulsion 8.3% (industrial condition),
• External coolant, emulsion 8.3%,
• Dry.

The axisymetric drilling axis is determined with a mechanical sensor on the sample. A milling step is performed on the top input face of the sample to avoid difficulties during the penetration of the drill bit. The measurement of temperature and force is launched just before the drilling test. The dynamometer Kistler 9125A measured the thrust force and the torque with an acquisition frequency of 1000 Hz. The evolutions of thrust force and torque allow highlighting the different stages of the drilling operation and thus identifying clearly a number of difficulties (chip evacuation, friction on margins…).

The thermocouples are placed at 5 mm depth from the input top surface, at the mid-thickness of the sample, and at 5 mm depth from the output surface. The most important information data are observed at mid-thickness of the sample because residual stresses will be measured at this location. It has been decided to avoid boundaries. In this location, two thermocouples are placed face to face at the same distance from the hole surface. The thermocouples next to the input and output surfaces are interesting to observe the boundary effects on heat transfer in the workpiece. Mineral-insulated thermocouples are introduced in the hole obtained by spark erosion machining. The thermocouples have a diameter of 0.75 mm, a type J, a sheath made of AISI304 and a hot junction grounded. To ensure a good conductivity between the thermocouple and the workpiece, a conductive glue is used (10 W/m/K). It is close to the thermal conductivity of 316L (16 W/m/K at 20 °C) compared with the low thermal conductivity of air (0.025 W/m/K at 20 °C).

**Fig. 1:**
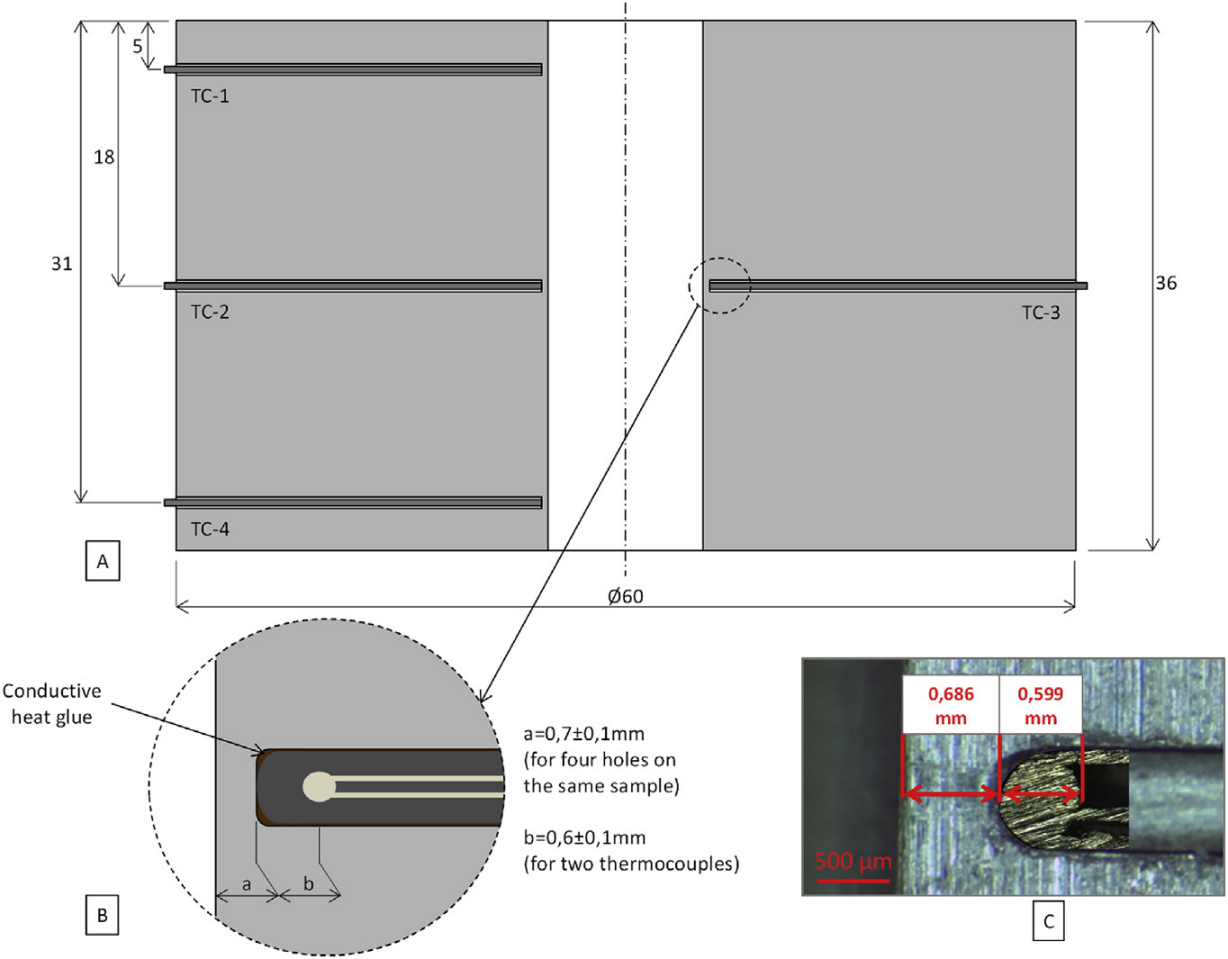
A Cross view of the workpiece with thermocouples positions – B Decomposition of distance between the hole and the hot junction of the thermocouple – C Example of a real decomposition of the distance between the hole and the hot junction of the thermocouple.

To identify the influence of lubrication on the temperature in the workpiece during the drilling operation, the temperature measurement location must be accurately known. The gap between the drilled surface and the temperature measurement location is composed of the gap between the drilled surface and the thermocouple endpoint and the gap between the thermocouple endpoint and the measurement location (Fig. 1). Special attention has been devoted to determining exactly the location of the thermocouple endpoint with regards to the drilled surface. The samples are cut and grinded until reaching the plane containing the axes of the thermocouple holes. Then the gap between the drilled surface and the endpoints of the thermocouples is measured with a binocular. The gap is about 0.7 mm (thermocouple diameter of 0.75 mm). A second step consists in studying the thermocouple that is cut, fixed in resin and polished to measure the gap between the endpoint of the thermocouple and the location of the temperature measurement. The gap measured is about 0.6 mm. The location of the temperature measurement is far from the endpoint of the thermocouple. Considering that they are located at the same place leads to an important error if experimental temperatures are used to determine the input heat flow on the surface or to check simulated temperatures. Finally, the gap between the drilled surface and the location of the temperature measurement is about 1.3 mm.

**Fig. 2:**
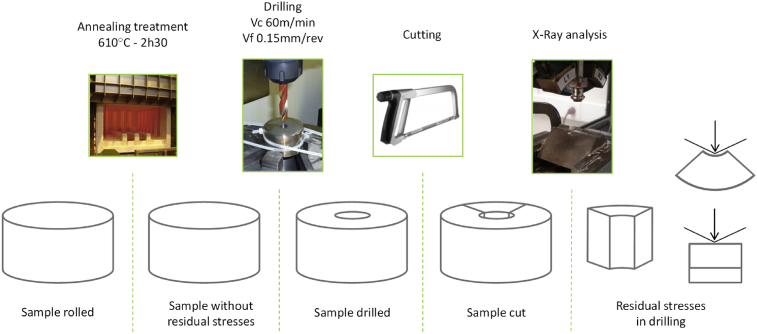
Process up to X-Ray analysis.

The X-Ray Diffraction technique is used to analyze residual stresses on the surface of the hole following the EN15305 standard [12]. To be able to analyze the inner surface of the hole with the X-ray diffraction device, the sample is cut. The influence of cutting on the residual stresses analyzed is discussed [13]. The process is presented in Fig. 2. The stress relaxation produced by the cutting is low and considered as negligible for the rest of the study.

## 3 Results

### 3.1 Mechanical phenomena

**Fig. 3:**
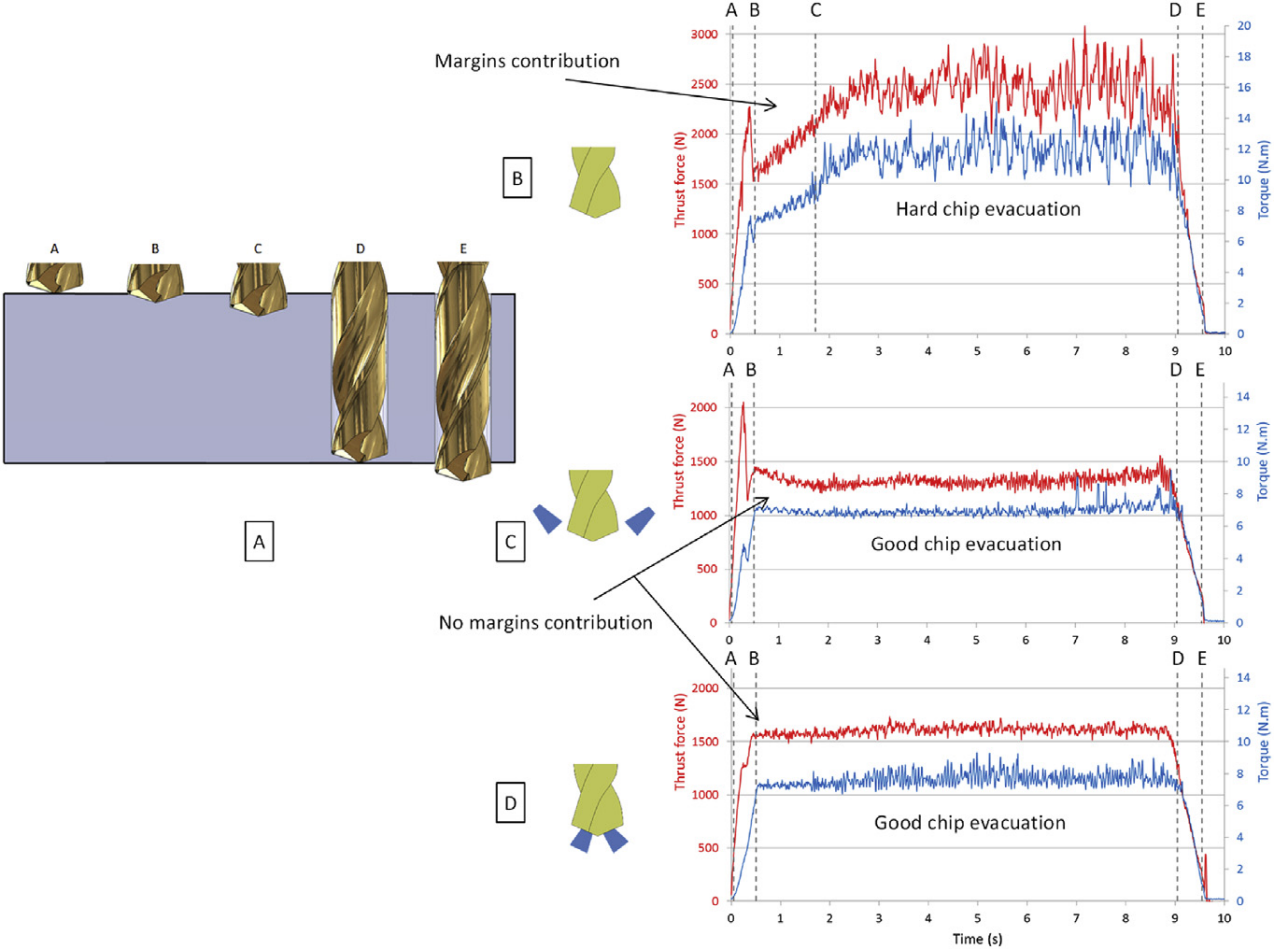
A Drill Positions in the hole – B Thrust force, torque in dry conditions – C Thrust force, torque with external lubrication – D Thrust force, torque with internal lubrication.

The observation of the thrust force and torque allows determining which part of the drill bit has an influence during the drilling operation (Fig. 3).

**Fig. 4:**
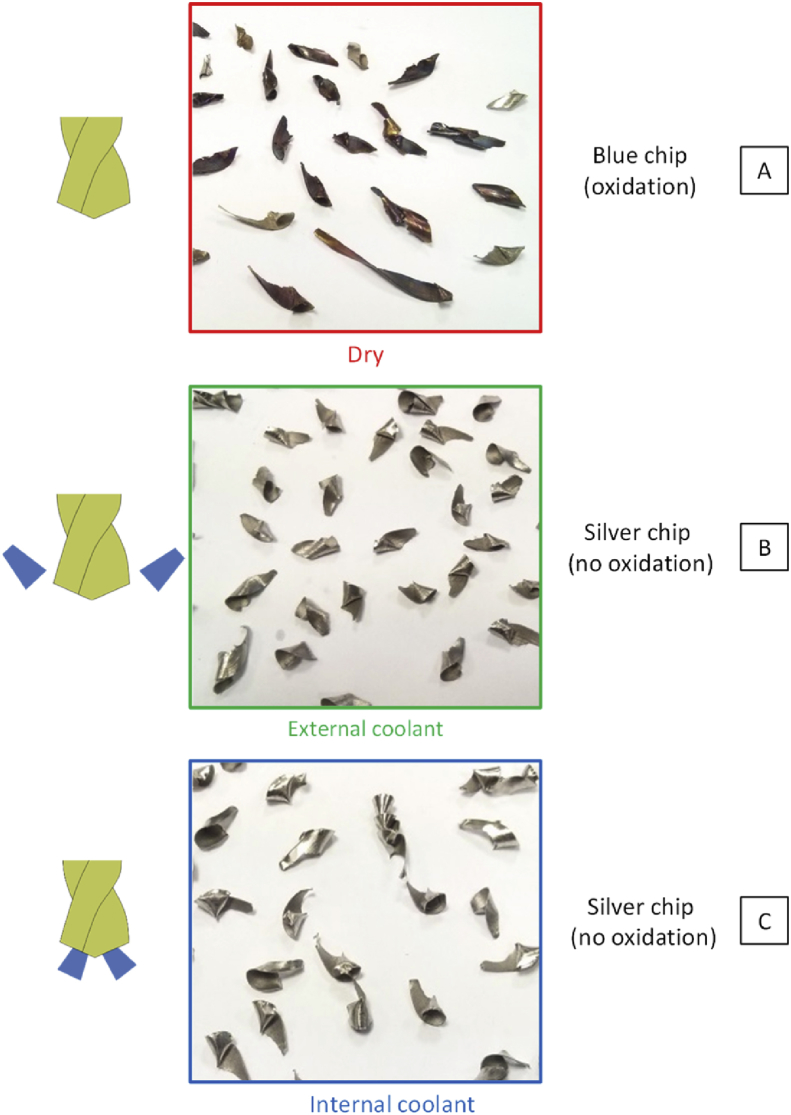
Chips after drilling for the three lubrication conditions – A Dry conditions – B External coolant – C Internal coolant.

For internal and external lubrication, the drilling operation is carried out without any difficulties. The force and torque remain steady during the process. During the dry test, the evolutions of the thrust force and torque highlight difficulties. After the input of the margins, the increase of the thrust force and torque indicate a friction increase. It leads to a heating of the workpiece and thus to thermal strains and constriction of matter on the drill. At the same time, the heating of the drill leads to expanding it. After several seconds, this phenomenon is steady and difficulties in the chip evacuation appear at the end of the drilling operation. The color and shapes of the chips demonstrate these difficulties (Fig. 4). In dry conditions, the chips are oxidized due to the important and localized heat generated during the cutting processes. The geometry is random due to the friction, the sticking and the crushing in the flute of the drill during the chip removal. In external and internal lubrication case, the friction and the heat generated are reduced. The remained heat is carried away by the coolant. Then the chip is fragmented easier. Finally, the color of the chip is the one expected without oxidation and the chips are short and similar.

**Fig. 5:**
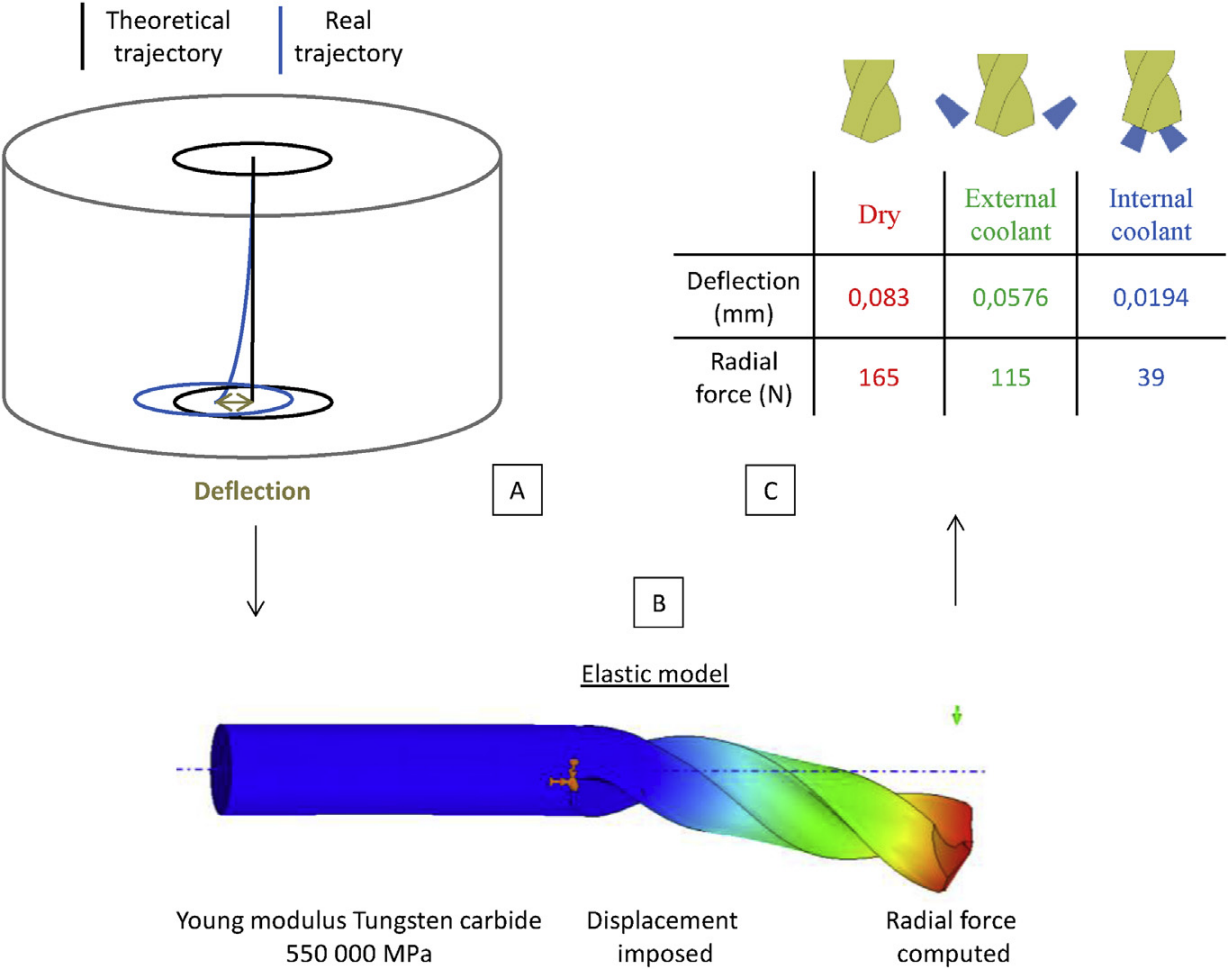
A Deflection measurement – B Elastic model to determine resultant force from measured deflection – C Radial force computed in three lubrication conditions.

The deflection is measured on the workpiece with a coordinate measuring machine and the radial force is deduced by means of an elastic FEM analysis. It has been shown in Fig. 5 that deflection increases when the use of coolant decreases. This is related to the low asymmetry of the drill geometry. This default is amplified with the drilling intensity. The computation of the radial forces leads to low values (39N - 115N - 165N) compared with the values of the thrust force (about 1500N) mainly due to the drill web. It is hard to detect which part of the thrust force has a mechanical impact on the drilled surface. In dry conditions, the contribution of margins leads to a variation of the thrust force of about 500N (from 1600 N to 2100 N). By considering a constant force along the lips, a torque of 8 N.m corresponds to a force of 667N along the lips. This part of the thrust force and torque inducing a mechanical impact on the drilled surface is not determined exactly but this estimation highlights the fact that the radial force due to deflection is not totally negligible. The radial force increases when the coolant is reduced, and the values determined could be used in modeling residual stresses.

### 3.2 Thermal phenomena

In machining, heat is generated in the primary, secondary and tertiary shearing areas. It is known that a large part of heat is carried away into the chip [14]. The study focuses on the drilled surface integrity, so it is important to determine the part of heat which reaches the final workpiece. The heat generated in the primary and tertiary shearing areas at the center of the drill is mostly carried away into the chip. The same phenomenon occurs with the heat generated at the contact between the drill and the chip. Clearly, the heat generated in the primary shearing area in the second half of the hole close to the wall hole has an important effect on the heat received by the workpiece. Temperatures in the workpiece and in the drill are dependent of the cutting conditions (drilling speed) and of the observation location as shown by Huang et al [15].

**Fig. 6:**
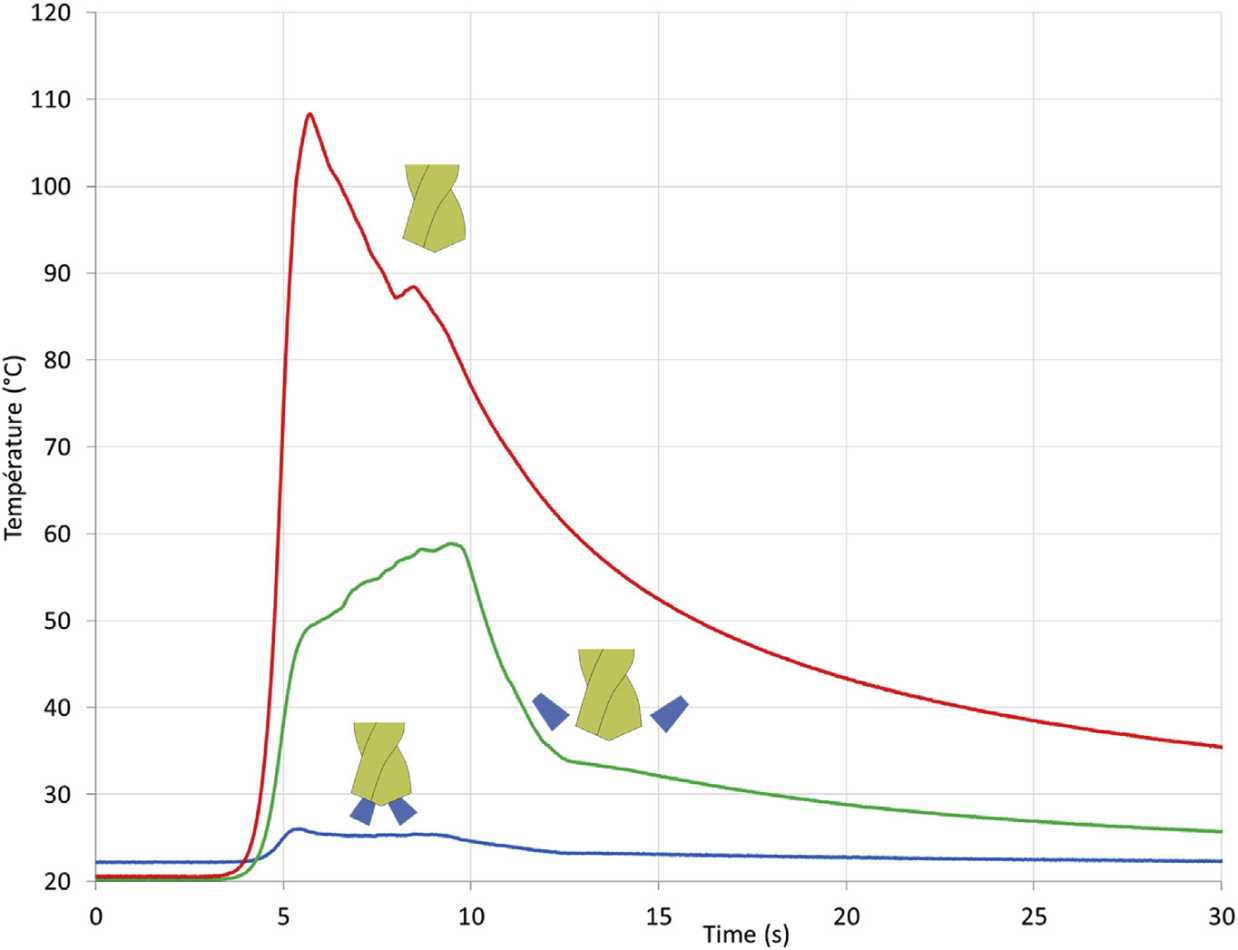
Influence of lubrication conditions on the heat transferred to the final workpiece.

The temperatures measured during the drilling tests (Fig. 6) highlight the influence of lubrication on the heat going to the workpiece. During dry drilling, the maximum temperature at 1.3 mm depth from the surface of the hole is equal to 110 °C. With the external coolant, a part of the heat generated is carried away by the fluid reducing the maximum temperature to 60 °C. With the internal coolant, the lubricant is forced to flow close to the cutting area corresponding to the heat generation area. The important velocity of the coolant leads to limiting the heat absorbed by the workpiece because the temperature does not increase significantly (about 5 °C).

For dry drilling, the value of 110 °C can be considered as low regarding the properties of the 316L steel and the analysis of the chips. However, the measurement is located at 1.3 mm depth from the drilled surface and the thermal gradient is significant. Therefore, a severe temperature must be reached in the cutting area and in the chip (between 600 and 800 °C) in agreement with the chip aspects.

**Fig. 7:**
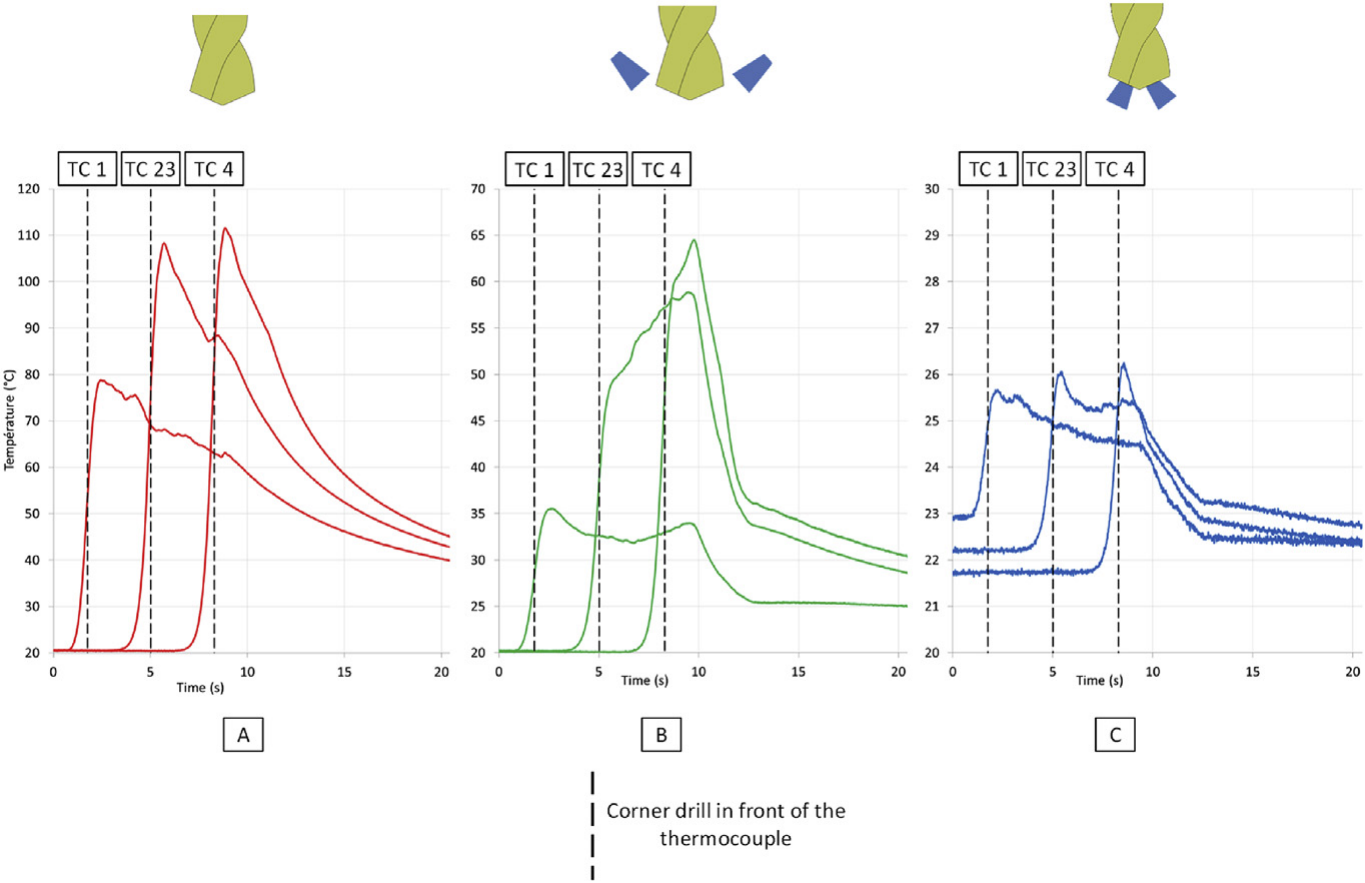
Temperatures for different locations on the sample (top, middle, bottom) – A Dry conditions – B External lubrication – C Internal lubrication.

The Fig. 7 shows the evolution of the temperature at each measurement location and for each lubrication condition. Regardless of drilling conditions, the temperature increases during the drilling operation. The drilled depth has an important influence on the temperature reached in the workpiece and thus, the surface integrity evolve along the drilling depth, it is demonstrated in an additional study [16]. At the end of the drilling operation, residual stresses are more tensile than at the beginning of the drilling.

For each thermocouple, a vertical bar is drawn on the graph to identify the instant when the drill corners are in front of the thermocouple. For each thermocouple, the maximum temperature is reached just after the drill goes away and the temperature begins to increase just before.

It is demonstrated that with the internal coolant, the workpiece is not thermally impacted compared to dry conditions.

### 3.3 Geometry

**Fig. 8:**
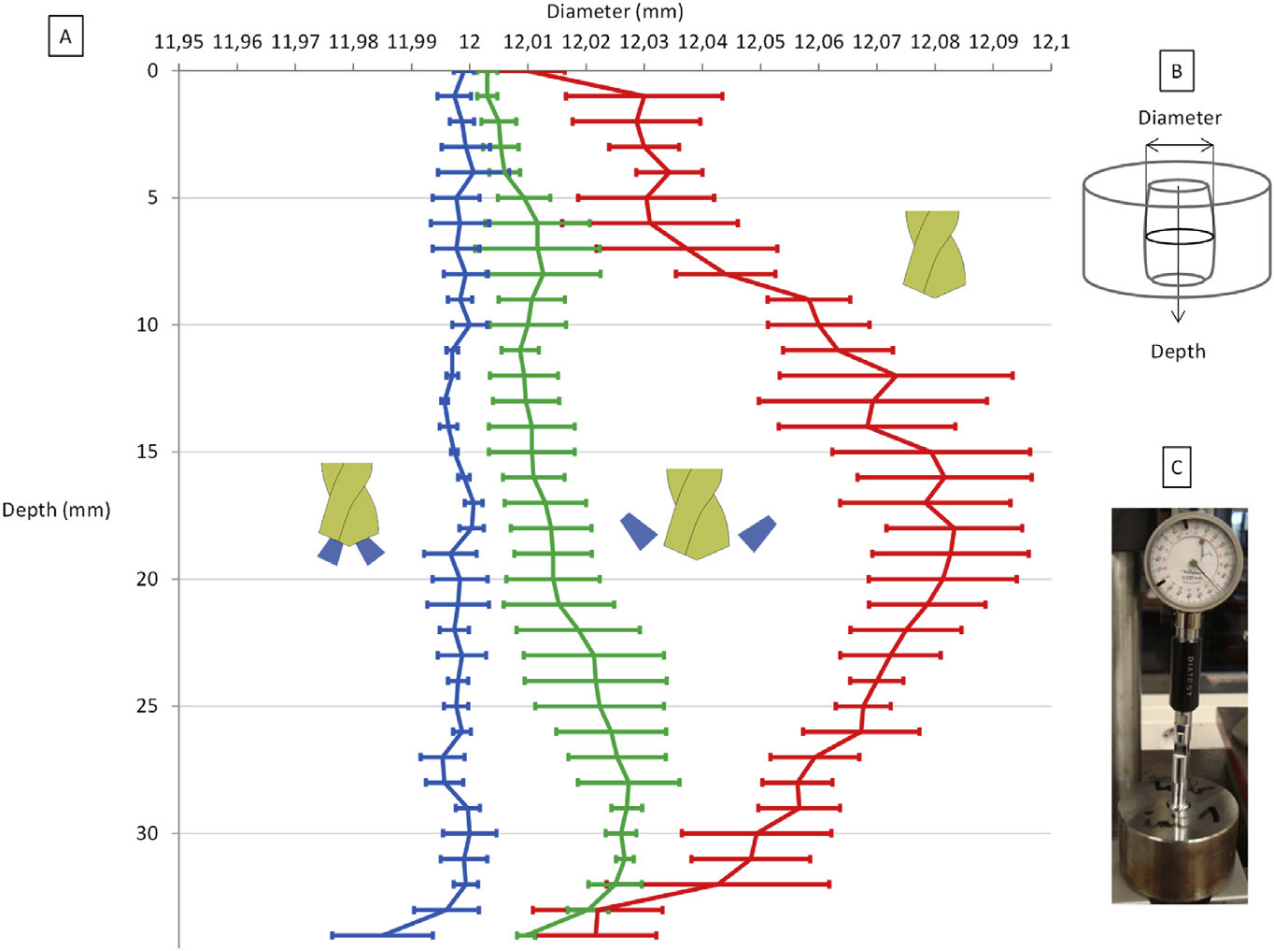
A Hole diameter measured in three lubrication conditions – B Principle of the diameter measurement of the hole – C Example of a diameter measurement with a diatest.

The hole diameter (Fig. 8) is measured with a diatest which allows getting an inscribed circle. The holes performed in the internal coolant keep a satisfactory diameter close to 12 mm from the input up to the end of the hole. For the external coolant, the difficulties to cool at the end of the drilling produce an increase in the diameter. In dry drilling, heat plays an important role, in the middle of the hole, the diameter is 0.1 mm greater. This highlights the fact that dry drilling does not provide a satisfactory shape of the hole. It is hard to explain the decrease in the hole diameter at the end of the drilling because the temperature measured at the last thermocouple location is higher than the temperature measured in the middle of the hole.

**Fig. 9:**
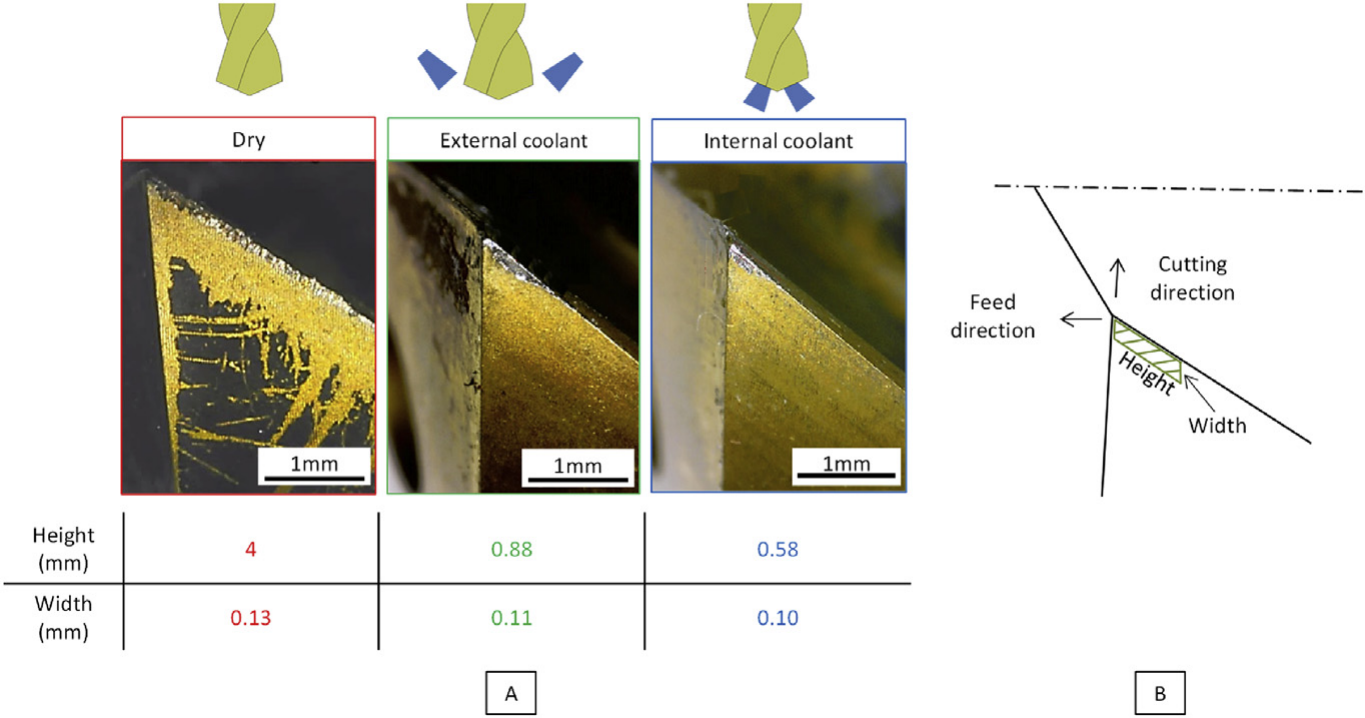
A Pictures of the friction area and values of height and width in three lubrication conditions – B Simplified representation of the friction area.

The thermal expansion of the drill and the thermal constriction of the workpiece provide a direct effect on the contact between the drill and the workpiece. The contact surface is identified in the three cases and the values of the height and width are retained (see Fig. 9). The heigth and the width are measured and presented. The width is not severely impacted by the lubrification condition (0.10 mm in internal coolant and 0.13 mm in dry condition) compared with the height (0.58 mm in internal coolant and 4 mm in dry condition). It is due to the drill geometry, indeed the clearance angle is larger in the cutting direction than in the feed direction, so during the thermal expansion, the matter is more easily in contact in the feed direction than in the cutting direction.

The contact surface is directly related to the temperature increase and the combination of the drill bit and workpiece expansion. The deflection can also contribute to the increase of the contact surface between the drill and the workpiece. Finally, the dry conditions are clearly severe and tend to increase the friction and thus the heat generated.

**Fig. 10:**
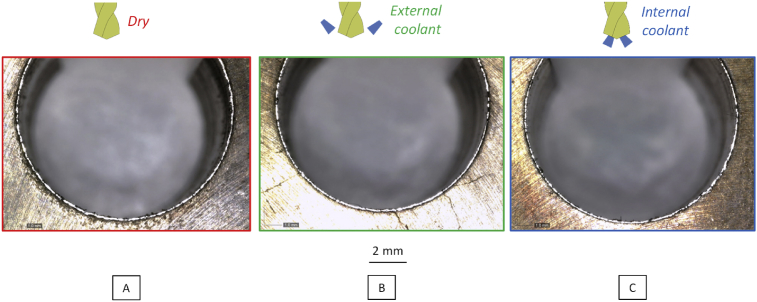
Picture of output drilled face to study burr formation – A Dry condition – B External lubrication – C Internal Lubrication.

Burrs after drilling in different lubrication conditions are similar (Fig. 10). Burrs are not as important as the ones presented by Segonds *et* al [17]. Pena *et* al [18] detect burr formation from the spindle torque. During our drilling tests, the torque analysis does not highlight significant burr formation. Guo and Dornfeld [19] present a finite element model to predict burr formation during drilling of 304L. Considering the small difference observed on the burrs in the three lubrication conditions, it is not necessary to proceed with any further analysis.

### 3.4 Surface integrity

**Fig. 11:**
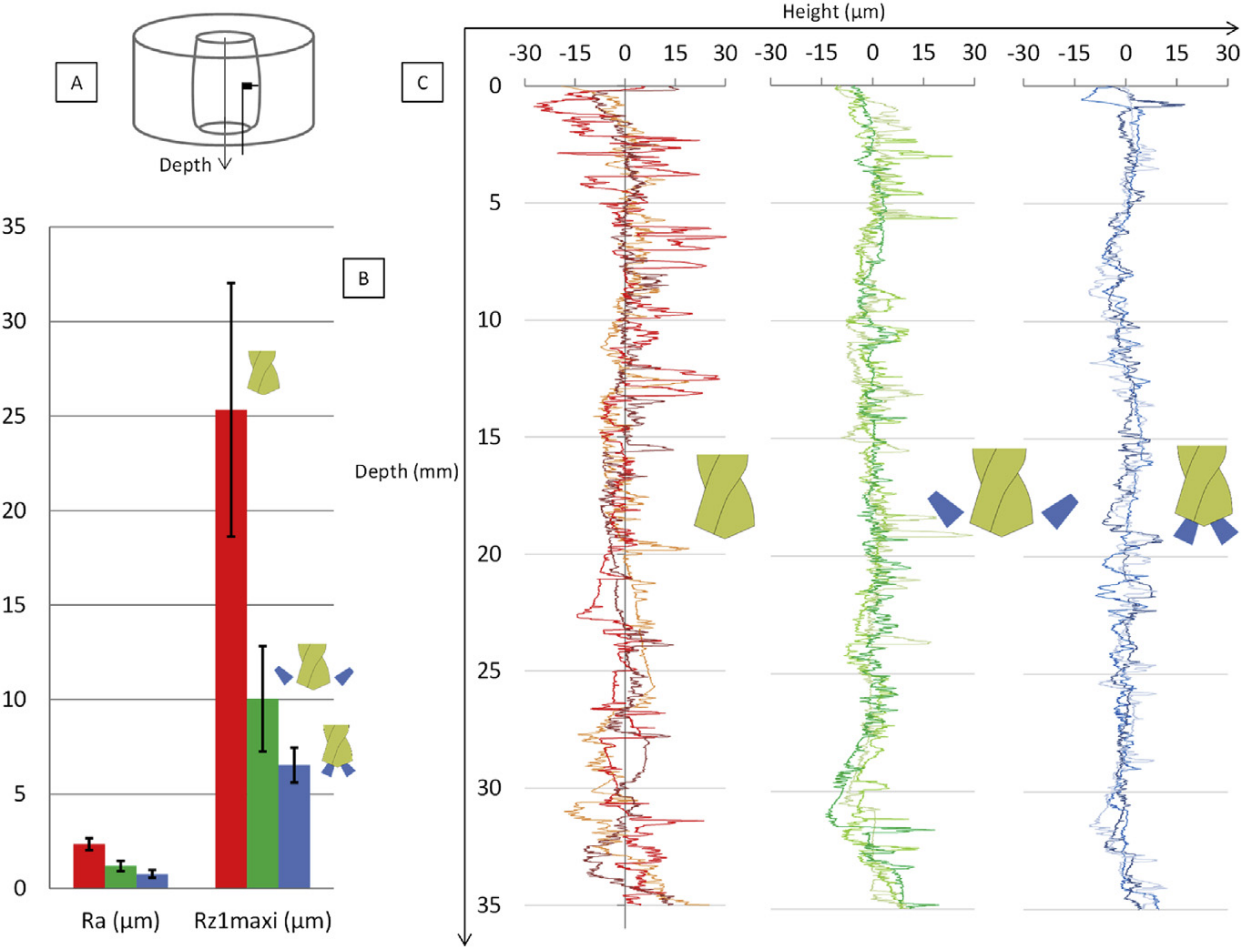
A Principle of the roughness measurement in the hole – B Values of Ra and RZ1maxi for the three lubrication conditions – C Profiles of roughness in three lubrication conditions.

Several roughness parameters are studied. Taylor-Hobson profilometer is used to measure the roughness on the surface of the drilled hole in the axis direction. A polynomial of degree 2 is taken off in order not to consider the incline and the curvature. Different parameters presented on the graph (Fig. 11) show a more significant roughness in dry conditions than in the external coolant conditions. The parameter R_z1_ is multiplied by 4 between the internal coolant and the dry condition. The internal coolant conditions give the best results. This is due to the chip temperature and the difficulties to evacuate it. It leads to chip sticking on the drilled surface or scratching. The roughness has an important effect on the crack generation and so on the fatigue lifetime.

**Fig. 12:**
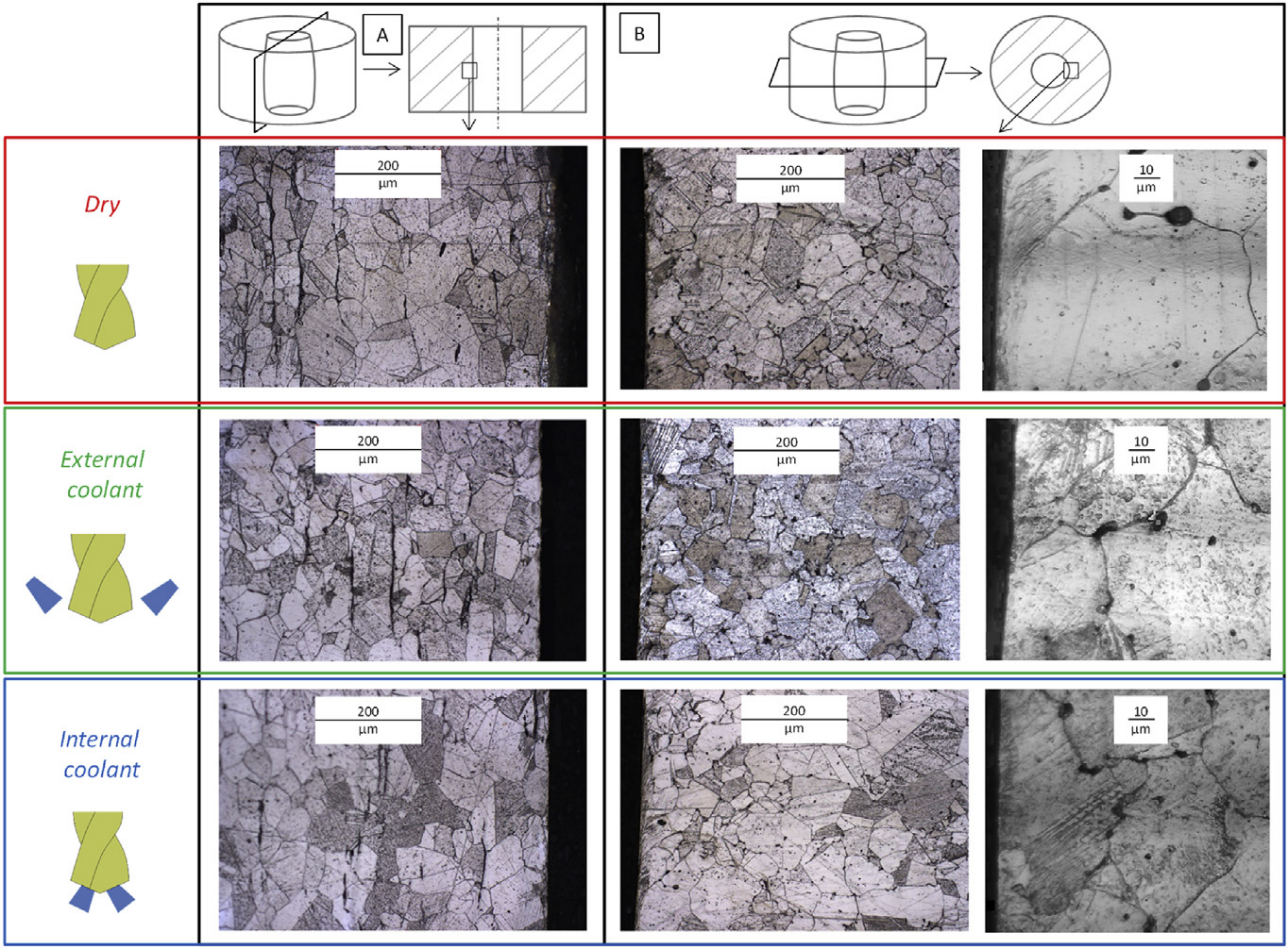
Microstructure close to the drilled surface – A Longitudinal cross section – B Transversal cross section.

The metallurgy in the longitudinal and transversal planes is studied at mid-depth of the hole, close to the area where residual stresses are analyzed. The 316L is an austenitic stainless steel. The Fig. 12 shows that no metallurgical change occurs during the drilling process. Indeed, the microstructure is similar in the base material and in the affected layer (100–200 μm under the drilled surface). No difference is identified between the three different lubrication conditions at this scale. This observation shows that the residual stresses analyzed in the three lubrication conditions are not caused by metallurgical changes. Only mechanical and thermal phenomena lead to the formation of residual stresses in the three lubrication conditions.

In the longitudinal plane, it is impossible to detect the feed direction due to the microstructure. In the transversal plane, the cutting direction can be identified with the grain orientation along the cutting direction. The grains close to the surface are deformed and cut but the size is not impacted by the cutting process.

**Fig. 13:**
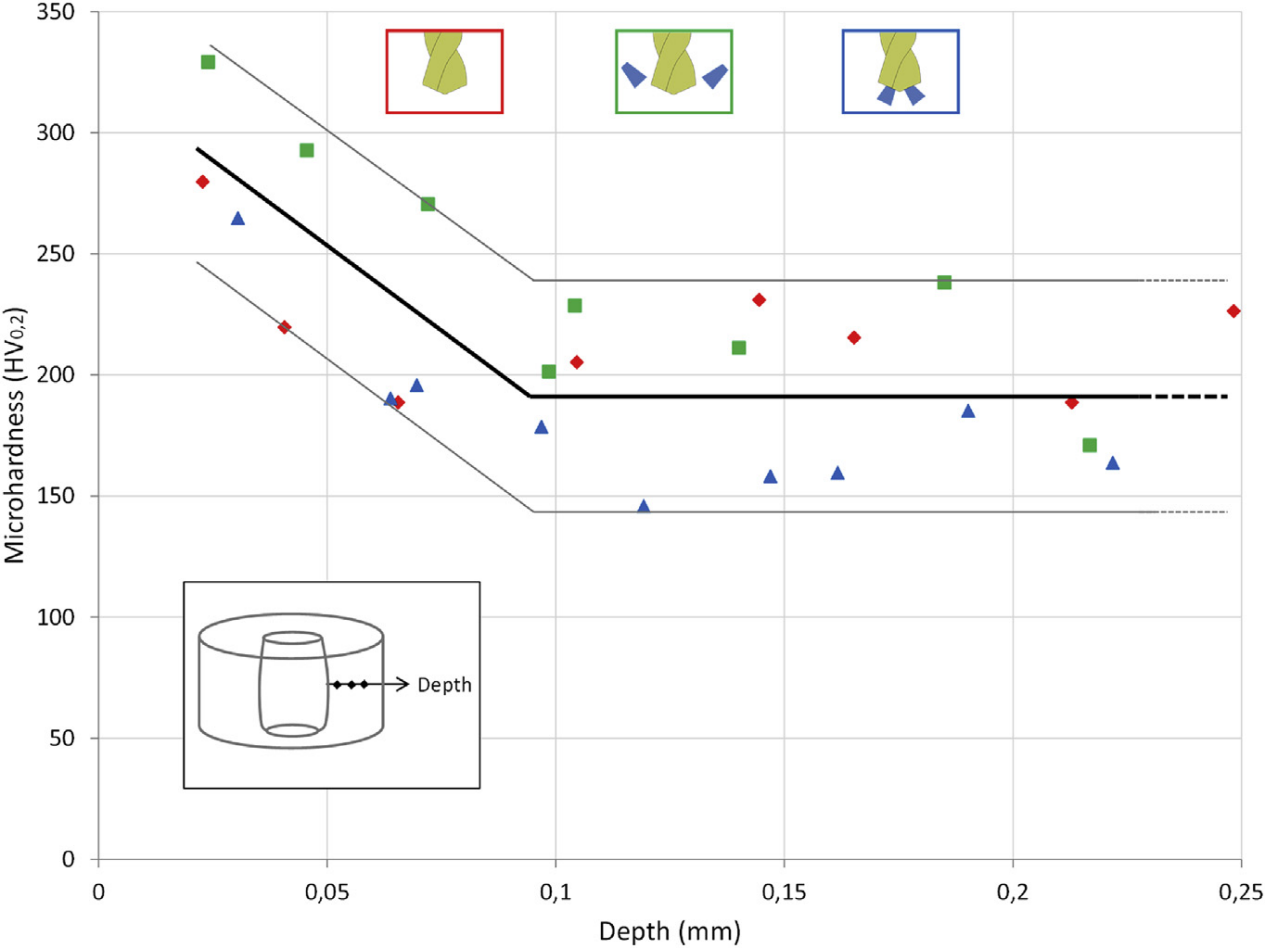
Microhardness.

The distribution of micro hardness is studied in Fig. 13. In the three lubrication cases (one sample studied for each lubrication condition), the micro hardness is high (280 HV) near the drilled surface and decreases up to 200 HV below 0.1 mm of depth. The increase of micro hardness near the surface is due to the mechanical effect of rubbing between the drill bit and the hole surface. The thermal softening, induced by an important heat that can generate a lower micro hardness than the value of the bulk material, is not identified in the dry condition.

**Fig. 14:**
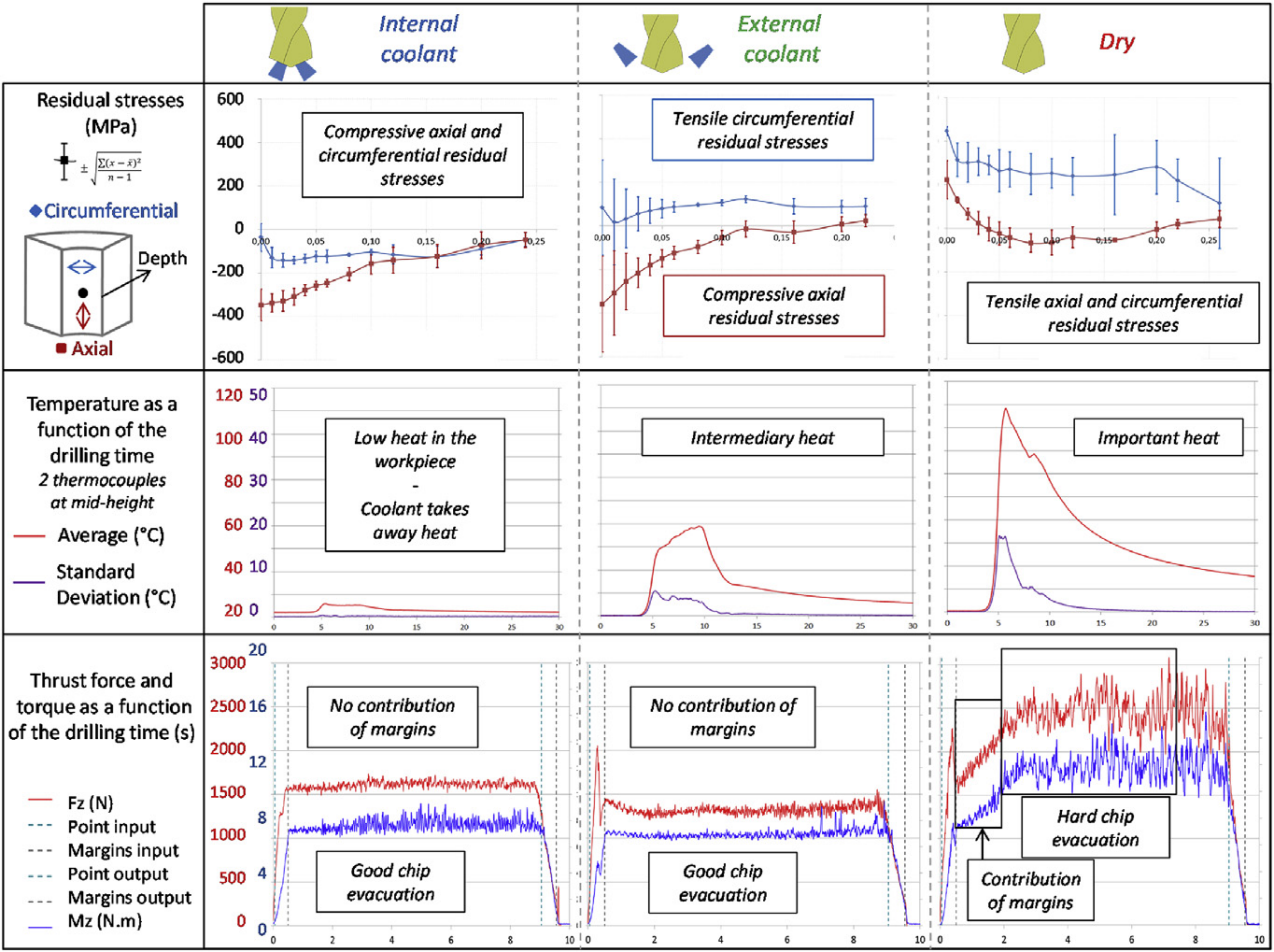
Summary of thermal and mechanical constraints imposed during drilling operations and residual stresses involved [16].

The residual stresses presented in Fig. 14 have a good repeatability for the three samples studied in each lubrication condition. In the internal coolant conditions, circumferential and axial residual compressive stresses are present with an affected layer of 0.1 mm. Drilling with an external coolant generates circumferential residual tensile stresses. Axial residual stresses seem like those in the internal coolant. The affected layer has also a 0.1 mm thickness. Finally, dry drilling provides two residual tensile stresses with high values (200 MPa in the axial direction and 400 MPa in the circumferential direction). The thickness of the affected layer increases by about 0.2 mm.

## 4 Discussion

This study reveals the influence of the lubrication conditions on the surface integrity during the drilling operation. The difficulties of dry drilling have a direct impact on the surface integrity. This difference is mainly due to the thermal phenomena coupled with tribological phenomena.

The first indicators of the drilling difficulties are the morphology of the chip, the thrust force and the torque measured during the drilling conditions. They are easily observable in an industrial environment and give information on a possible weakness of the surface integrity. The contact surface is also an indicator of the drilling conditions. An important contact surface and the material deposition on the drill mean that drilling has been performed in severe conditions.

Then the second indicator of the cutting conditions is the temperature. It must be measured close to the drilled surface to clearly identify the difference between the different lubrication cases. It is less easy to measure the temperature correctly and to determine exactly the measurement location. A method is presented to determine it. The temperature measurement is necessary to identify the difference between the internal and external lubrication conditions because this cannot be observed by means of the chip morphology and the force measurement.

With lubrication, an important part of the heat is removed by the lubricant. In dry conditions, the heat is not removed and the contact conditions between the drill and the material are severe. This explains why the heat received by the workpiece is higher.

After the analysis of the drilling operation conditions, this study focuses on the quality of the drilled workpiece. The diameter of the hole is clearly not respected in dry conditions and some indicators show that the surface integrity is disturbed when the lubrication is reduced. Concerning the roughness and the residual stresses, the internal lubrication corresponds to the best condition and dry drilling to the worst.

In drilling, the roughness is mainly the result of the chip evacuation conditions and of the contact condition between the tip of the tool and the surface. In lubrication conditions, the chip is cooled and breaks easily. Shorter chips can be easily evacuated. In dry conditions, chips are longer. Then the surface of the hole generated is softened by the high temperature and can be easily scratched during the chip evacuation.

Residual stresses are the result of strong plastic strain gradients in the material. In dry conditions, significant heat leads to a significant expansion near the hole surface and thus to a high plastic strain gradient between the surface and the rest of the workpiece [20]. This explains why a high level of tensile residual stresses is analyzed in dry conditions. The internal lubrication corresponds to a drilling process with a final hole surface receiving a low heat. The mechanical phenomena near the tip of the drill are more important than the thermal aspects and lead to compressive residual stresses. Finally, the case under external lubrication conditions is a compromise between the thermal and mechanical phenomena. It shows that the residual stresses in the circumferential direction easily tend to tensile stresses (compared with internal coolant conditions) whereas the axial residual stresses require a higher level of temperature to tend to tensile residual stresses (reached only in dry conditions).

The micro hardness highlights the hardening in surface due to mechanical effects and a thermal softening is not identified in dry conditions. As far as the microstructure is concerned, the grain orientation along the cutting direction is identified.

## 5 Conclusion

Different lubrication conditions in the drilling process are studied by considering the drilling of a 316L austenitic stainless steel. The lubrication has a significant effect on the drilling conditions and on the quality of the drilled hole.

Several indicators are used to determine the link between the mechanical and thermal drilling phenomena and the surface integrity of the drilled hole.

The main results are:

- The indicators studied (chip, forces and temperatures) show a drilling operation which is severe in dry conditions, mainly in the last third of the depth.
- Difficulties during the drilling operation influence the hole quality (geometry and surface integrity).
- In dry conditions, the important amount of heat received by the final workpiece leads to tensile residual stresses. In external conditions, the amount of heat received is lower, only circumferential residual stresses are tensile. In internal lubrication conditions, the heat generated is carried away with the coolant, mechanical phenomena become preponderant and the residual stresses are compressive.
- The dry condition is the worst condition considering surface integrity. The roughness is high, and the surface residual stresses are tensile. Thus, the crack generation is favored. The affected layer is large and residual stresses are also tensile in depth, the conditions are combined to continue the crack propagation and to lead to the part breakage.

In demanding industrial environments such as aeronautics or energy, lubrication systems must be clearly controlled.

## Declarations

### Author contribution statement

Mathieu Girinon: Performed the experiments; Analyzed and interpreted the data; Wrote the paper.

Habib Karaouni, Erwan Jourden: Analyzed and interpreted the data; Wrote the paper.

Ugo Masciantonio, Fabien Lefebvre: Performed the experiments; Wrote the paper.

Frédéric Valiorgue, Joël Rech: Conceived and designed the experiments; Wrote the paper.

Eric Feulvarch: Contributed reagents, materials, analysis tools or data; Wrote the paper.

### Funding statement

This work was supported by CETIM, AREVA and SAFRAN.

### Competing interest statement

The authors declare no conflict of interest.

### Additional information

No additional information is available for this paper.
